# Relevance of QuantiFERON-TB Gold Plus and Heparin-Binding Hemagglutinin Interferon-γ Release Assays for Monitoring of Pulmonary Tuberculosis Clearance: A Multicentered Study

**DOI:** 10.3389/fimmu.2020.616450

**Published:** 2021-02-02

**Authors:** Carole Chedid, Eka Kokhreidze, Nestani Tukvadze, Sayera Banu, Mohammad Khaja Mafij Uddin, Samanta Biswas, Graciela Russomando, Chyntia Carolina Díaz Acosta, Rossana Arenas, Paulo PR. Ranaivomanana, Crisca Razafimahatratra, Perlinot Herindrainy, Julio Rakotonirina, Antso Hasina Raherinandrasana, Niaina Rakotosamimanana, Monzer Hamze, Mohamad Bachar Ismail, Rim Bayaa, Jean-Luc Berland, Flavio De Maio, Giovanni Delogu, Hubert Endtz, Florence Ader, Delia Goletti, Jonathan Hoffmann

**Affiliations:** ^1^ Laboratoire des Pathogènes Emergents, Fondation Mérieux, Centre International de Recherche en Infectiologie, INSERM U1111, Lyon, France; ^2^ Département de Biologie, Ecole Normale Supérieure de Lyon, Lyon, France; ^3^ National Center for Tuberculosis and Lung Diseases (NCTBLD), Tbilisi, Georgia; ^4^ International Centre for Diarrhoeal Disease Research, Bangladesh (icddr,b), Dhaka, Bangladesh; ^5^ Instituto de Investigaciones en Ciencias de la Salud, National University of Asunción, Asunción, Paraguay; ^6^ Hospital General de San Lorenzo, MSPyBS, Asunción, Paraguay; ^7^ Institut Pasteur de Madagascar, Antananarivo, Madagascar; ^8^ Centre Hospitalier Universitaire de Soins et Santé Publique Analakely (CHUSSPA), Antananarivo, Madagascar; ^9^ Laboratoire Microbiologie, Santé et Environnement (LMSE), Doctoral School of Sciences and Technology, Faculty of Public Health, Lebanese University, Tripoli, Lebanon; ^10^ Dipartimento di Scienze di Laboratorio e Infettivologiche, Fondazione Policlinico Universitario “A. Gemelli”, IRCCS, Rome, Italy; ^11^ Dipartimento di Scienze biotecnologiche di base, cliniche intensivologiche e perioperatorie – Sezione di Microbiologia, Università Cattolica del Sacro Cuore, Rome, Italy; ^12^ Fondation Mérieux, Lyon, France; ^13^ Service des Maladies Infectieuses et Tropicales, Hospices Civils de Lyon, Lyon, France; ^14^ Translational Research Unit, Department of Epidemiology and Preclinical Research, “L. Spallanzani” National Institute for Infectious Diseases (INMI), IRCCS, Rome, Italy

**Keywords:** tuberculosis, immunomonitoring, interferon-gamma release assays, heparin-binding haemagglutinin adhesin, QuantiFERON, treatment monitoring, inflammatory markers

## Abstract

**Background:**

Tuberculosis (TB) is a leading infectious cause of death. To improve treatment efficacy, quicker monitoring methods are needed. The objective of this study was to monitor the response to a heparin-binding hemagglutinin (HBHA) interferon-*γ* (IFN-*γ*) release assay (IGRA) and QuantiFERON-TB Gold Plus (QFT-P) and to analyze plasma IFN-*γ* levels according to sputum culture conversion and immune cell counts during treatment.

**Methods:**

This multicentered cohort study was based in Bangladesh, Georgia, Lebanon, Madagascar, and Paraguay. Adult, non-immunocompromised patients with culture-confirmed pulmonary TB were included. Patients were followed up at baseline (T0), after two months of treatment (T1), and at the end of therapy (T2). Clinical data and blood samples were collected at each timepoint. Whole blood samples were stimulated with QFT-P antigens or recombinant methylated *Mycobacterium tuberculosis* HBHA (produced in *Mycobacterium smegmatis;* rmsHBHA). Plasma IFN-*γ* levels were then assessed by ELISA.

**Findings:**

Between December 2017 and September 2020, 132 participants completed treatment, including 28 (21.2%) drug-resistant patients. rmsHBHA IFN-*γ* increased significantly throughout treatment (0.086 IU/ml at T0 *vs*. 1.03 IU/ml at T2, p < 0.001) while QFT-P IFN-*γ* remained constant (TB1: 0.53 IU/ml at T0 *vs*. 0.63 IU/ml at T2, p = 0.13). Patients with low lymphocyte percentages (<14%) or high neutrophil percentages (>79%) at baseline had significantly lower IFN-*γ* responses to QFT-P and rmsHBHA at T0 and T1. In a small group of slow converters (patients with positive cultures at T1; n = 16), we observed a consistent clinical pattern at baseline (high neutrophil percentages, low lymphocyte percentages and BMI, low TB1, TB2, and MIT IFN-*γ* responses) and low rmsHBHA IFN-*γ* at T1 and T2. However, the accuracy of the QFT-P and rmsHBHA IGRAs compared to culture throughout treatment was low (40 and 65% respectively). Combining both tests improved their sensitivity and accuracy (70–80%) but not their specificity (<30%).

**Conclusion:**

We showed that QFT-P and rmsHBHA IFN-*γ* responses were associated with rates of sputum culture conversion. Our results support a growing body of evidence suggesting that rmsHBHA IFN-*γ* discriminates between the different stages of TB, from active disease to controlled infection. However, further work is needed to confirm the specificity of QFT-P and rmsHBHA IGRAs for treatment monitoring.

## Introduction

Tuberculosis (TB) is one of the leading causes of death by infectious disease in the world, causing 1.5 million deaths in 2019 (1). The treatment of pulmonary TB requires antibiotic multitherapies that last at least six months (2, 3) and can have toxic side effects. Consequently, treatment adherence is not optimal, especially in primary care settings (4, 5). Currently, anti-TB treatment monitoring relies on *Mycobacterium tuberculosis* (*M. tuberculosis*) detection by sputum smear microscopy and culture when possible (6). Sputum culture is the gold standard, but it is slow and requires high biosafety laboratory environments (7), while smear microscopy is highly sample- and operator-dependent and has poor sensitivity (8, 9). There is a clinical need for quicker anti-TB treatment monitoring tests adapted to primary care settings (10), that require accessible samples (blood, urine, feces) and limited laboratory equipment (11).

QuantiFERON-TB Plus (QFT-P; Qiagen) is an ELISA-based IFN-*γ* release assay (IGRA) that tests for exposure to *M. tuberculosis.* While it is useful for the triage of suspected TB patients, it cannot discriminate between active and latent TB (12) and has shown little clinical relevance for TB treatment monitoring so far (10). Previous works highlighted a general decrease in IFN-*γ* levels across TB treatment (13–18), and a study on QuantiFERON Gold In-Tube highlighted the presence of downregulated non-TB specific IFN-*γ* responses (Mitogen tube) were associated with poor treatment outcomes (19). However, persistently high quantitative results as well as heterogeneous QFT-P conversion rates make the test unlikely to be adapted for individual treatment monitoring (20–22).

Recently, the use of QFT-P in combination with the detection of IFN-*γ* responses to recombinant *Mycobacterium smegmatis* heparin-binding hemagglutinin (hereafter called “rmsHBHA IGRA”) as an additional stimulation antigen has shown promise to stratify TB cases by stage of infection and progression to disease (23–27), and to monitor TB treatment outcomes (28). In particular, negative or low levels of IFN-*γ* production in response to rmsHBHA stimulation have been associated with active TB disease as opposed to latent infection. However, this assay has been explored only in studies in non-TB endemic settings, or with no drug-resistant TB patients.

The primary objective of this prospective multicentered cohort study was to monitor the plasma IFN-*γ* response to rmsHBHA and QFT-P antigens during anti-TB treatment. Moreover, recent data collected in the same cohort highlighted an association between baseline circulating white blood cells (WBC) and TB treatment outcome (29); hence, a secondary objective was to describe rmsHBHA and QFT-P IFN-*γ* values in subsets of patients stratified according to demographics, immune cell counts, and culture conversion during treatment. For that purpose, we conducted a cohort study in five countries with low- or middle income status and high- or middle TB incidence rates (30) (Bangladesh, Georgia, Lebanon, Madagascar, and Paraguay), focusing on adult, HIV-uninfected, culture confirmed drug-susceptible or drug-resistant pulmonary TB patients.

## Materials and Methods

### Study Design and Sample Population

This descriptive study was nested within a multicenter prospective cohort study assessing the prognostic value of blood-based immunological markers for TB treatment monitoring. The study was based in five institutions from the Mérieux Foundation GABRIEL network (31), with the approval of national TB programs and the following ethical committees: the international center for diarrheal diseases and research, Bangladesh (icddr,b) in Dhaka, Bangladesh; the National Center for Tuberculosis and Lung Diseases (NTCLD) in Tbilisi, Georgia; the *Laboratoire Microbiologie, Santé et Environnement (LMSE, Université Libanaise)*, in Tripoli, Lebanon; the *Institut Pasteur de Madagascar* in Antananarivo, Madagascar; and the *Instituto de Investigationes en Ciencias de la Salud (Universidad Nacional de Asunción; IICS-UNA)* in Asunción, Paraguay. All recruited patients provided written informed consent and standard biosecurity and institutional safety procedures were followed in all study sites.

### Cohort Recruitment, TB Diagnosis, and Patient Follow-Up

The sample size was evaluated to detect a difference in rmsHBHA IFN-*γ* between baseline and end of treatment, with the following assumptions: we aimed for a level of significance of 95% and a power of 80%, assuming a minimum average expected difference of 1.6 IU/ml in rmsHBHA IFN-*γ* levels throughout treatment based on reported estimates (32), with an expected standard deviation of 3 IU/ml at each repeated measurement. We calculated (33) that a sample size of 117 was required to reach significance. As this study was nested in a cohort study with a sample size of 200, we aimed to enroll more patients to account for missing data. Patients were recruited if diagnosed with microbiologically confirmed pulmonary TB (positive culture and/or sputum smear and/or GeneXpert). Patients with HIV or diabetes mellitus and children under 15 years were excluded. In downstream analyses, patients under immunocompromising treatment, patients with negative cultures at inclusion, and patients who were lost-to-follow-up were excluded. Detailed procedures for microbiological diagnosis, drug sensitivity testing, and therapeutic regimen composition were described previously (29).

Patients were followed up: at inclusion (T0), after two months of treatment (T1), at the end of therapy [T2; 6 months for drug-susceptible (DS-TB) patients, nine to 24 months for drug-resistant (DR-TB) patients]. Data were presented for all patients followed up until T2 at least. Patients were on Directly Observed Treatment (DOT) and received treatment according to standard protocols (2, 3, 34). In this study, culture conversion at T1 was used to define three patient subsets: fast converters (definitive culture conversion between T0 and T1), slow converters (definitive culture conversion between T1 and T2), and patients with poor treatment outcome (positive culture results at T2: treatment failure; or positive culture at T3: relapse).

### On-*S*ite Whole Blood Collection and Cell Count

At every follow-up visit, 10 ml of whole blood were drawn: 4 ml was used for other downstream analyses, 1 ml was collected in EDTA tubes and used to measure whole blood cell counts by standardized automated systems available in the study sites as listed previously (29), and 5 ml was used for *in vitro* blood stimulation. For the QFT-P assay, 1 ml of whole blood was seeded directly into each of four QuantiFERON-TB Gold Plus (QFT-P, Qiagen) tubes as per the manufacturer’s instructions. The NIL tube contained no antigens and was used as a negative control. The TB1 and TB2 QFT-P tubes are coated with commercial *M. tuberculosis*-specific antigenic peptide pools. TB1 tubes contain two mycobacterial peptides, ESAT-6 (>15aa) and CFP-10 (8–13aa), which elicit specific immune responses from CD4+ T lymphocytes (35). TB2 tubes contain an additional commercial peptide pool designed to induce CD8+ T lymphocyte stimulation. MIT tubes are coated with commercial phytohemagglutinin-like bacterial antigens and were used as a positive control (35). For the rmsHBHA assay, 1 ml of blood was seeded into a NIL tube which was complemented with rmsHBHA (provided by the Delogu laboratory, UNICATT, Rome, Italy (23)), at a final concentration of 5 µg/ml. Within 2 h of blood collection, samples were placed at 37°C in a 5% CO_2_ atmosphere and incubated for 24 h. After incubation, plasmas were separated from the cell fraction by decantation, and stored at −80°C until further use.

### I*nterferon*-γ Release Assay

IFN-*γ* secretion was quantified using the QFT-P ELISA kit (Qiagen) according to the manufacturer’s instructions. Briefly, plasma samples were thawed at room temperature, and 50 µl of plasma was tested. Optical density results were compared to log-normalized values from freshly reconstituted IFN-*γ* kit standards. To account for potential immunomodulation phenomena unrelated with TB treatment, baseline IFN-*γ* level values (NIL tubes) were subtracted from antigen-stimulated IFN-*γ* values (MIT, TB1, TB2, rmsHBHA). According to the kit’s sensitivity range, the maximum for IFN-*γ* level values was set at 10 IU/ml and negative values were rescaled to 0.

### Assay Comparability Between Study Sites

To optimize comparability, a sample handling and processing protocol common to all study sites was developed, and on-site trainings were performed to standardize experimental processes such as instrument settings and timings. A tracking sheet was developed and used to follow sample shipment and standardize storage conditions in all sites. As the models of measurement instruments used in the different sites could not be homogenized, instrument readings were tested with QuantiFERON Control Panel (Qiagen) prior to launching the project. Finally, internal controls were added to each ELISA run to control for experimental variation and verify storage quality. Briefly, whole blood from a healthy donor was collected and stimulated with QFT-P antigens following the same protocol as described previously. Plasma was then separated and aliquoted and added to each ELISA run.

### Clinical Data Collection and Statistical Analysis

Standardized clinical report and data collection forms were implemented to ensure dataset homogeneity as described previously (29). Data were entered into the CASTOR database system (Version 1.4, Netherlands) (36), and cleaned and analyzed in R (version 3.6.2). Discrete variables were analyzed using Fisher’s Exact test with Bonferroni’s *post-hoc* test (37). Normal, continuous variables were analyzed with Student’s t-test. Non-normal, continuous variables were analyzed with the Mann–Whitney test, or the Kruskal–Wallis rank sum test with Dunn’s Kruskal–Wallis Multiple Comparisons *post-hoc* test (38). Repeated measures of non-independent continuous variables were analyzed using the Friedman rank sum test, with the Wilcoxon–Nemenyi–McDonald-Thompson *post-hoc* test (39). As the HBHA IGRA was not commercialized and QFT-P was designed to screen latent rather than active TB patients, we used Receiver Operating Curve (ROC) analyses to identify optimal IFN-*γ* thresholds adapted for this cohort, discriminating culture positive from culture negative patients. The overall QFT-P test was considered positive if either TB1 or TB2 was above their respective thresholds. ROC analyses and logistic regression were performed as described previously (29). In particular, multivariate logistic regression analyses were adjusted with the combination of variables that minimized the Akaike Information Criterion (AIC) for most tested predictors, while including important adjustment variables (age, sex, drug sensitivity, country).

## Results

### Sociodemographic, Clinical, and Microbiological Characteristics of the Cohorts

**Figure 1:**
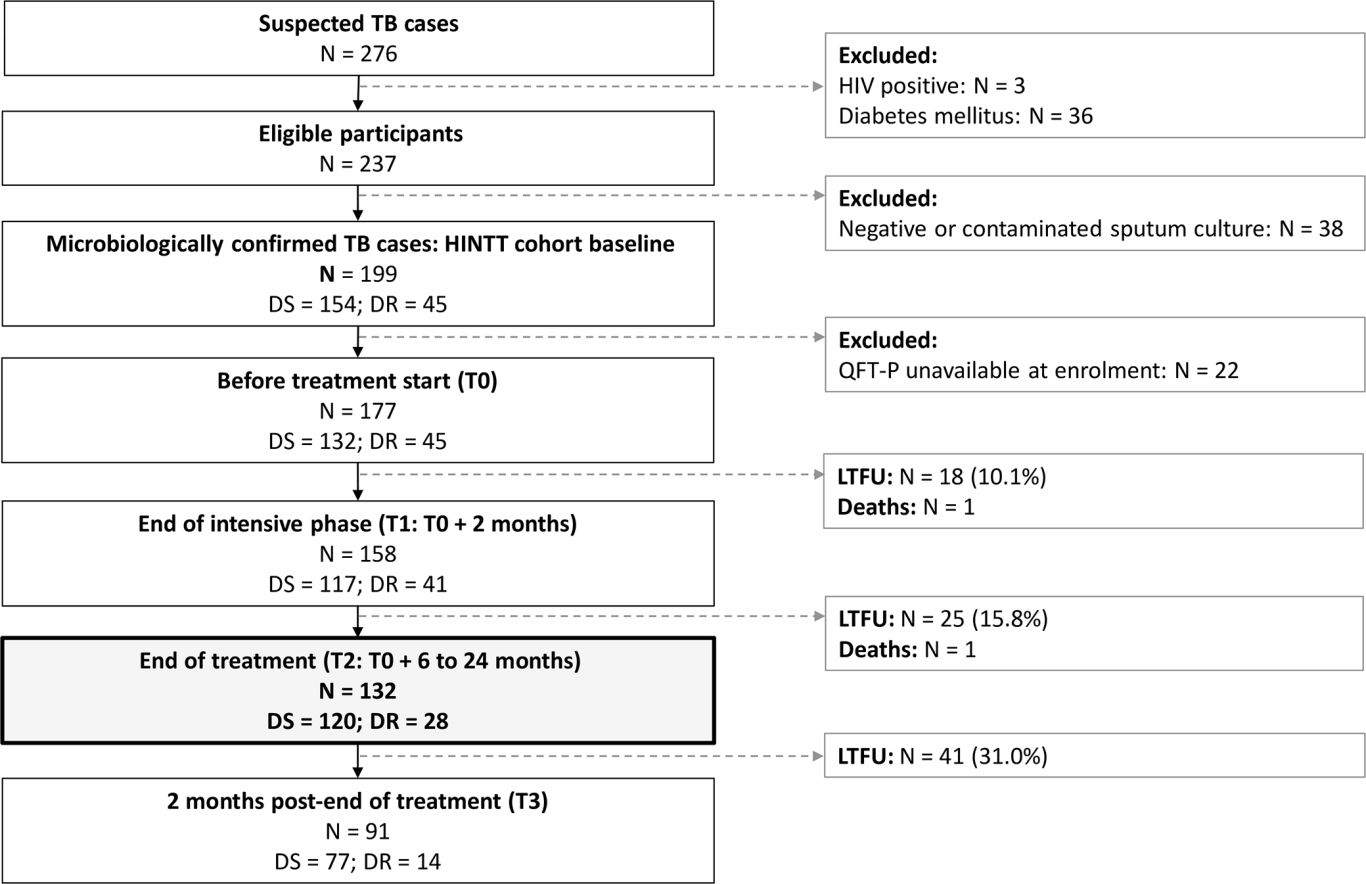
Patient inclusions between December 2017 and September 2020. DR, drug-resistant; DS, drug-susceptible; LTFU, lost to follow-up. TB, tuberculosis. HIV, human immunodeficiency virus. Treatment for DS-TB patients lasted 6 months. Treatment for DR-TB patients lasted 9 to 24 months.

Between December 2017 and September 2020, 199 eligible patients with culture confirmed active pulmonary TB were recruited in Dhaka (Bangladesh), Tbilisi (Georgia), Tripoli and Akkar (Lebanon), Antananarivo (Madagascar), and Asunción (Paraguay). As of September 2020, 132 of them were followed at least until the end of treatment and had available IGRA data (**Figure 1**). Among these patients, 21.2% (28/132) were diagnosed with DR-TB. The sociodemographic characteristics of DS-TB and DR-TB patients were similar at inclusion (**Table 1**). Sociodemographic characteristics were compared between study sites, and significant differences were observed concerning age, BMI at inclusion, and BCG vaccination rates (**Supplementary Table 1**). All enrolled patients were sputum culture positive at inclusion. Most patients were also positive for sputum smear microscopy (sensitivity: 78.0%, 103/132) and/or GeneXpert (98.4%, 125/132). Three (3.9%) cases of treatment failure and one (0.7%) case of relapse were recorded (**Table 1**).

**Table 1:** Sociodemographic and clinical characteristics of drug-susceptible and drug-resistant patients at inclusion.

N	ALL	DS-TB	DR-TB	*p*
	132	104	28	
***Patient demographics***				
Age (years)	27 (21–36.2)	27 (21–37.2)	27.5 (19.7–33.2)	*0.41*
Sex (male)	62.9% (83/132)	64.4% (67/104)	57.1% (16/28)	*0.51*
Treatment outcome				
*Cured and completed*	95.5% (126/132)	94.2% (98/104)	100% (28/28)	*0.34*
*Completed*	1.5% (2/132)	1.9% (2/104)	0	*-*
*Failure*	2.3% (3/132)	2.9% (3/104)	0	*-*
*Relapse*	0.8% (1/132)	1% (1/104)	0	*-*
Country of origin				
*Bangladesh*	28.8% (38/132)	20.2% (21/104)	60.7% (17/28)	***>0.001***
*Georgia*	23.5% (31/132)	20.2% (21/104)	35.7% (10/28)	*0.13*
*Lebanon*	5.3% (7/132)	6.7% (7/104)	0	*0.34*
*Madagascar*	27.3% (36/132)	34.6% (36/104)	0	***>0.001***
*Paraguay*	15.2% (20/132)	18.3% (19/104)	3.6% (1/28)	*0.073*
BMI at inclusion	18.7 (16.9–21.3)	18.83 (16.9–21.4)	18.7 (17.5–21.0)	*0.79*
WBC absolute count at inclusion (cells/mm^3^)	9745 (7365–12032)	9695 (7350–12055)	10150 (7725–11850)	*0.65*
Neutrophils at inclusion (% of WBC)	75 (68–79.1)	75 (68.3–79)	78 (66.7–80.2)	*0.34*
Lymphocytes at inclusion (% of WBC)	18 (14–25)	18 (14–25)	17 (13.7–24.5)	*0.52*
Monocytes at inclusion (% of WBC)	4.4 (2–7)	5 (2–8.0)	4 (3–5.2)	*0.42*
Number of household contacts	4 (3–6)	4 (3–6)	4 (3.75–6.2)	*0.86*
BCG vaccination	86.2% (94/109)	86.4% (76/88)	85.7% (18/21)	*1*
***Risk factors and comorbidities***				
Smoking	46.2% (61/132)	46.2% (48/104)	46.4% (13/28)	*1*
Alcohol abuse	22% (29/132)	24% (25/104)	14.3% (4/28)	*0.57*
Injectable drug use	3.8% (5/131)	2.9% (3/104)	7.4% (2/27)	*0.27*
Jail detention history	8.5% (11/130)	9.8% (10/102)	3.6% (1/28)	*0.57*
Chronic HCV infection	1.6% (2/129)	2% (2/101)	0	*0.75*
Other disease^1^	6.2% (7/113)	7.8% (7/90)	0	*0.34*
***History of TB***				
Prior exposure to active TB patients	29% (38/131)	29.1% (30/103)	28.6% (8/28)	*0.23*
Documented previous TB episode	15.1% (20/132)	11.5% (12/104)	28.5% (8/28)	***0.048***
***Previous TB outcome***				
Cured and completed	61.1% (11/18)	61.5% (8/13)	60% (3/5)	*1*
Treatment completed	11.1% (2/18)	7.7% (1/13)	20% (1/5)	*0.49*
Outcome not evaluated or unknown	16.7% (3/18)	15.4% (2/13)	20% (1/5)	*1*
Treatment failure	11.1% (2/18)	15.4% (2/13)	0	*-*

Data are given as % (N) or median (interquartile range). DS-TB, drug-susceptible tuberculosis; DR-TB, drug-resistant tuberculosis; BMI, body mass index; WBC, white blood cells; 1: asthma, hypertension, inflammation. P-values are given for DS-TB vs. DR-TB.

No patients had HIV, non-TB chronic pulmonary diseases, renal diseases, solid tumors, chronic HBV infection, were pregnant, or under immunosuppressive therapies (corticosteroids, calcineurin inhibitors, biologics).

### Dynamics of Interferon-γ Levels During Treatment and Influence of Sociodemographic Factors

**Figure 2:**
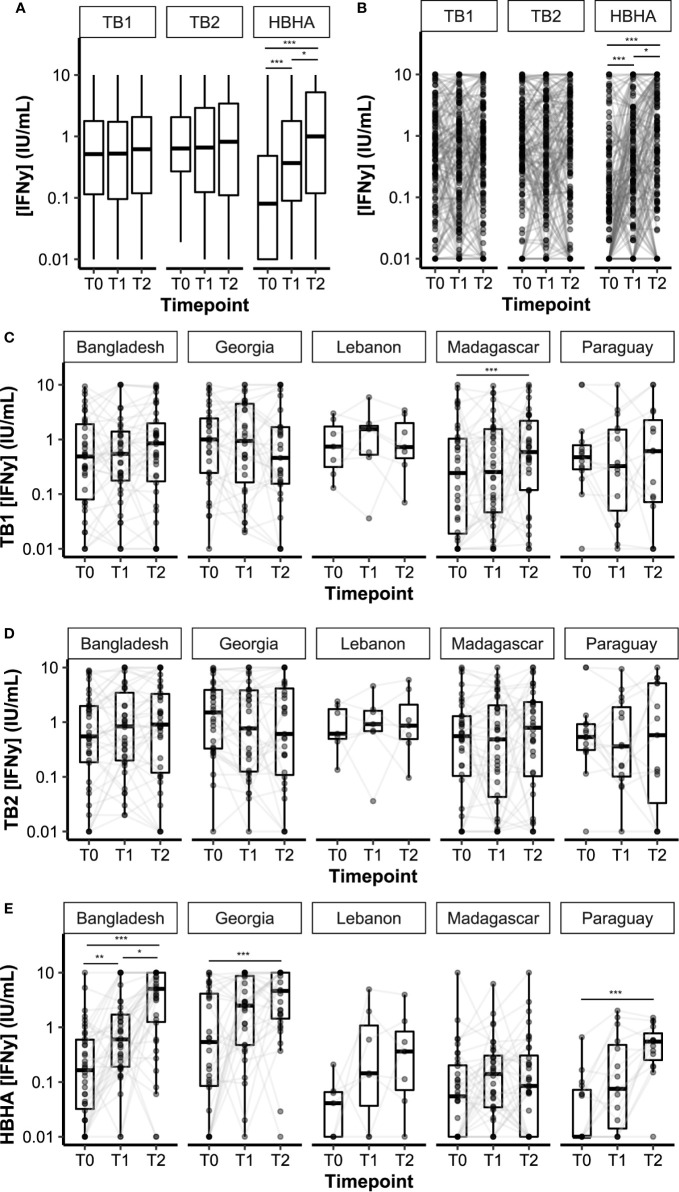
Dynamics of plasma IFN-γ response to QFT-P and HBHA stimulations over the course of TB therapy. Data are given as median + interquartile range. Each black dot represents one patient at one timepoint. Grey lines connect data points from the same patient. **(A)** Median IFN-γ responses in the complete cohort (n = 132 per timepoint). **(B)** Individual IFN-γ responses in the complete cohort. **(C–E)** Stratification per study site. Bangladesh (n = 38), Georgia (n = 31), Lebanon (n = 7), Madagascar (n = 36), Paraguay (n = 20). T0: baseline. T1: baseline + 2 months. T2: end of treatment. Data were compared using Friedman’s Exact Test with the Wilcoxon–Nemenyi–McDonald-Thompson *post-hoc*, or the Mann–Whitney U test **(B)**. *p < 0.05; **p < 0.01; ***p < 0.001.

Plasma IFN-*γ* levels in response to TB1, TB2, or HBHA stimulations were measured during anti-TB treatment (**Figure 2**). The median IFN-*γ* response to TB1 and TB2 remained constant over time, while the median response to rmsHBHA increased significantly (**Figure 2A**). Individual IFN-*γ* levels were heterogeneous in all three stimulation conditions (**Figure 2B**). To account for individual variations, rmsHBHA/TB1 and rmsHBHA/TB2 IFN-*γ* ratios were evaluated, and a significant increase in both ratios was still observed overall (**Supplementary Figures 1A–C**). We also measured the TB2-TB1 IFN-*γ* response, as a proxy for the QFT-P CD8^+^ T-cell response (**Supplementary Figure 1D**). No significant difference was detected over time. The impact of sociodemographic parameters on IFN-*γ* levels was assessed but no significant association was detected (data not shown).

Overall, QFT-P positivity rates remained constant during treatment (T0 *vs*. T2: 52 vs. 55%, p = 0.71), whereas rmsHBHA positivity rates increased significantly (T0 *vs*. T2: 31 *vs*. 67%, p < 0.001 (**Table 2**). We also calculated the slopes of rmsHBHA and QFT-P IFN-*γ* variations during treatment (**Table 2**). An increased INF-*γ* response to TB1, TB2, and rmsHBHA was observed in 55.3% (73/132), 56.8% (75/132), and 77.3% (102/132) of patients respectively.

**Table 2:** Qualitative evolution of QFT-P and HBHA IFN-*γ* levels during treatment.

Positivity rate at each timepoint	HBHA	TB1 only	TB2 only	QFT-P (TB1 and/or TB2)
T0	31.1% (41/132)	4.5% (6/132)	34.8% (46/132)	52.3% (69/132)
T1	56.1% (74/132)	2.3% (3/132)	42.4% (56/132)	50.8% (67/132)
T2	67.4% (89/132)	2.3% (3/132)	43.9% (58/132)	55.3% (73/132)
**Trend between T0 and T1**				
Increase	66.7% (88/132)	56.1% (74/132)	47.7% (63/132)	–
Decrease	24.2% (32/132)	41.7% (55/132)	47.7% (63/132)	
Constant	9.1% (12/132)	2.3% (3/132)	4.5% (6/132)	
**Trend between T0 and T2**				
Increase	77.3% (102/132)	55.3% (73/132)	56.8% (75/132)	**-**
Decrease	15.2% (20/132)	40.9% (54/132)	41.7% (55/132)	
Constant	7.6% (10/132)	3.8% (5/132)	1.5% (2/132)	

Data are given as % (N). T0, baseline; T2, end of treatment; QFT-P, QuantiFERON-TB Gold Plus; Constant, no difference in IFN-y levels between T0 and T2, regardless of variations during treatment. Positivity was set at 0.75 IU/ml for QFT-P and at 0.22 IU/ml for HBHA based on ROC analyses.

IFN-*y* levels over time were then stratified per study site (**Figures 2C–E**). Similar trends were observed in all cohorts for TB1 and TB2 IFN-*γ* levels, except in the Madagascar site in which an increase in TB1 IFN-*γ* was recorded between T0 and T2. Variation in IFN-*γ* levels produced by rmsHBHA-stimulated samples was different between study sites: similar in increase and order of magnitude in the Bangladesh and Georgia cohorts on the one hand, as well as in Paraguay and Lebanon on the other hand; however, no increase was observed in the Madagascar cohort, as well as lower IFN-*γ* values (**Supplementary Table 2**). Mitogen IFN-*γ* levels were also significantly lower in the Madagascar cohort than in the Georgia cohort at all timepoints (**Supplementary Table 2**).

### Effect of Neutrophil and Lymphocyte Percentages on Interferon-γ Release Assay Interferon-γ Response During Treatment

**Figure 3:**
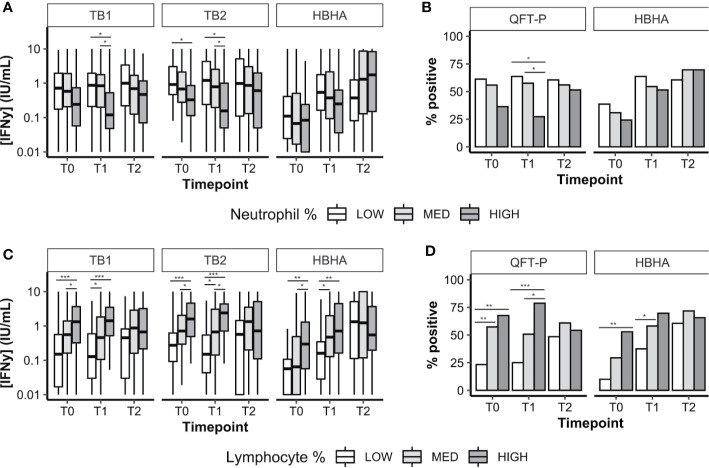
Plasma IFN-γ response to TB-specific QFT-P antigens or HBHA stimulation in patients stratified by WBC counts over the course of TB therapy. **(A, C)** Quantitative IFN-γ response. Data are given as median + interquartile range and were compared using Kruskal–Wallis’ test with Dunn’s *post-hoc* when necessary. **(B, D)** Data were given as a percentage of each group and were compared using Fisher’s Exact Test with Bonferroni’s correction when necessary. Positivity was set at 0.75 IU/ml for QFT-P TB1, 0.71 IU/ml for QFT-P TB2 (the overall QFT-P result was positive if TB1 and/or TB2 were positive) and at 0.22 IU/ml for HBHA, based on ROC analyses. Low, medium, and high WBC or lymphocyte groups were defined as follows: low: <1^st^ quartile; medium: 1^st^–3^rd^ quartiles; high: >3^rd^ quartile. WBC, white blood cells; T0, baseline; T1, baseline + 2 months; T2, end of treatment; *p < 0.05; **p < 0.01; ***p < 0.001.

We analyzed the distribution of neutrophil percentages, and stratified IFN-*γ* results according to three groups: low neutrophils (less than the first quartile), intermediate neutrophils (between first and third quartiles), and high neutrophils (**Figure 3A**; threshold values are available in **Supplementary Table 3**). Similar analyses were performed with lymphocyte percentages (**Figure 3C**). We also evaluated the proportion of QFT-P and rmsHBHA positivity at each timepoint, stratified by neutrophil (**Figure 3B**) and lymphocyte percentages (**Figure 3D**). As HBHA stimulation was not performed using a commercial kit, Receiver Operating Curve (ROC) analyses were performed to identify the optimal rmsHBHA IFN-*y* threshold value differentiating culture-positive patients from culture-negative patients at any timepoint. The resulting Area Under the Curve (AUC) was maximized for an IFN-*y* cutoff value of 0.24 IU/ml (AUC 0.725, 95% CI 0.674–0.777). Overall, neutrophil and lymphocyte percentages directly impacted IFN-*γ* responsiveness to TB-specific antigens: QFT-P and rmsHBHA IFN-*y* levels and positivity rates were significantly higher in patients with low neutrophil (**Figures 3A, B**) or with high lymphocyte proportions (**Figures 3C, D**). This statistically significant trend was also observed when comparing the subgroup of patients with both low neutrophil and high lymphocyte percentages to the rest of the cohort (data not shown).

### Effect of the Culture Conversion Status at 2 Months on the Interferon-γ Release Assay Interferon-γ Response Throughout Treatment

**Figure 4:**
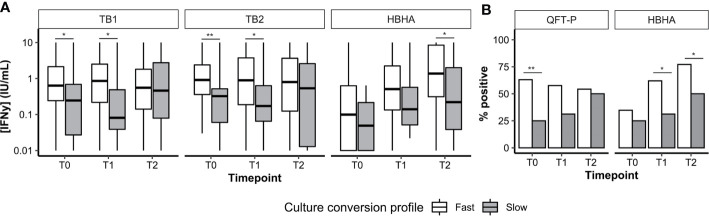
Culture conversion profiles and plasma IFN-*y* dynamics. Plasma IFN-γ levels (**A**, represented as medians + interquartile range) and IGRA positivity rates **(B)**. T0, enrolment; T1, T0 + 2months; T2, end of treatment; Fast, conversion between T0 and T1 (n = 92). Slow, conversion between T1 and T2 (n = 16). *p < 0.05; **p < 0.01 (Mann–Whitney U Test or Fisher’s Exact Test).

Overall, 112 patients had available culture data at T0, T1, and T2. Most patients were fast converters (definitive culture conversion between T0 and T1; 82.1%, 92/112) or slow converters (definitive culture conversion between T1 and T2; 14.2%, 16/112). Poor treatment outcomes were recorded in four patients (treatment failure, 2.7%, 3/112; relapse, 0.9%, 1/112; data not shown). Among successfully cured patients (n = 108), median IFN-*γ* levels (**Figure 4A**) as well as QFT-P and rmsHBHA IGRA positivity rates (**Figure 4B**) were stratified according to the culture conversion profiles. In slow converters, TB1 and TB2 IFN-*γ* levels at T0 and T1 and rmsHBHA IFN-*γ* levels at T2 were significantly lower than in fast converters. Similarly, QFT-P positivity rates at T1 and rmsHBHA positivity rates at T1 and T2 were significantly lower in slow converters.

Then, we calculated the sensitivity, specificity, and accuracy of the QFT-P and rmsHBHA IGRAs for TB treatment monitoring at T1 and T2, using culture as a reference standard (**Supplementary Table 4**). At T1 and T2 respectively, the accuracy of the QFT-P IGRA was of 44 and 46%, and the accuracy of TB2-TB1 was of 52 and 55%. For the rmsHBHA IGRA, we evaluated the test performances of negative rmsHBHA results (*i.e.* rmsHBHA IFN-*γ* ≤ 0.22 IU/ml), since lower rmsHBHA IFN-*y* values were observed before treatment. The accuracy of the rmsHBHA IGRA was of 64 and 65% at T1 and T2, respectively. Finally, we generated a score which was positive when the QFT-P result was positive and the rmsHBHA result was negative. The sensitivity of this combined score was of 86% at T1 and 82% at T2, and its accuracy reached 77% at T1 and 81% at T2, but its specificity remained inferior to 30% at both timepoints. Similar results were observed with a score combining rmsHBHA and TB2-TB1.

### Association Between White Blood Cell Counts, Culture Conversion, and Interferon-γ Release Assay Interferon-γ Response During Treatment

We compared the immune cell counts (**Supplementary Table 5**) and the baseline sociodemographic characteristics (**Supplementary Table 6**) of patients according to their culture conversion profiles. No difference was detected between slow and fast converters for immune cell counts, but at T0 and T1, patients with treatment failure or relapse had significantly higher neutrophil percentages (at T0, median 84%, interquartile range (IQR) 81.5–86.5; at T1, 79%, IQR 75–81.75), and lower lymphocyte percentages (at T0, 12.5%, IQR 9.2–15.2; at T1, 15.5%, IQR 11–21.2) than successfully treated patients. The BMI at inclusion was significantly lower in slow than in fast converters, and slower conversion rates were observed in the Madagascar cohort.

Then, logistic regression analyses were performed to identify associations between slow culture conversion and immune cell counts or IGRA results (**Table 3**). In univariate analyses, significant associations were detected between slow conversion and MIT IFN-*γ* at T0 (odds ratio (OR) 0.78, p = 0.001) and T1 (OR 0.84, p = 0.021), with QFT-P IGRA positivity at T0 (OR 0.19, p = 0.008), and with rmsHBHA IGRA positivity at T1 (OR 0.24, p = 0.015) and T2 (OR 0.29, p = 0.029). The BMI at inclusion was also associated (OR 0.791, p = 0.025).

**Table 3:** Associations between time to culture conversion, IFN-*γ* response, and selected clinical parameters.

Parameter	Timepoint	Descriptive analysis (n = 108)			Univariate analysis		Multivariate analysis^1^			
		Slow responders (n = 16; reference)	Fast responders (n = 92)	*P*	OR (95%CI)	*p*	aOR (95%CI)	*p*	C	AIC
MIT IFN-*γ* (IU/ml)	*T0*	4.9 (1.8–10)	10 (9.82–10)	*0.0028*	0.78 (0.67–0.91)	*0.001*	0.65 (0.44–0.86)	*0.009*	0.75	57.5
	*T1*	8.3 (3.9–10)	10 (9.93–10)	*0.0039*	0.84 (0.72–0.98)	*0.021*	0.77 (0.56–1.01)	*0.076*	0.68	63.9
	*T2*	9.4 (4.8–10)	10 (9.94–10)	*0.023*	0.88 (0.76–1.04)	*0.11*	0.78 (0.57–1.05)	*0.10*	0.66	64.7
Positive QFT-P IGRA	*T0*	25% (4/16)	63% (58/92)	*0.006*	0.19 (0.051–0.61)	*0.008*	0.045 (0.002–0.35)	*0.013*	0.77	57.6
	*T1*	31.2% (5/16)	56.5% (52/92)	*0.10*	0.33 (0.099–0.99)	*0.059*	0.39 (0.064–1.97)	*0.27*	0.64	66.2
	*T2*	50% (8/16)	53.3% (49/92)	*0.98*	0.88 (0.29–2.57)	*0.81*	3.27 (0.59–24.1)	*0.20*	0.65	65.7
Positive HBHA IGRA	*T0*	25% (4/16)	35.9% (33/92)	*0.57*	0.62 (0.16–1.96)	*0.44*	0.39 (0.075–1.71)	*0.24*	0.64	66.7
	*T1*	31.2% (5/16)	65.2% (60/92)	*0.013*	0.24 (0.071–0.73)	*0.015*	0.076 (0.003–0.67)	*0.045*	0.74	61.9
	*T2*	50% (8/16)	77.2% (71/92)	*0.033*	0.29 (0.097–0.89)	*0.029*	0.79 (0.17–4.13)	*0.77*	0.61	67.1
Lymphocyte % of WBC	*T0*	17.5 (12.8–19.5)	19.0 (15–26)	*0.099*	0.93 (0.84–1.00)	*0.086*	0.94 (0.83–1.05)	*0.33*	0.64	66.4
	*T1*	23.0 (16.2–28.0)	25.0 (20.7–31)	*0.099*	0.93 (0.86–0.99)	*0.052*	0.92 (0.83–0.99)	*0.078*	0.68	63.3
	*T2*	29.5 (23.5–36.2)	30.0 (25.9–36)	*0.93*	1.01 (0.95–1.06)	*0.76*	1.01 (0.94–1.08)	*0.89*	0.62	67.5
Body mass index	*T0*	17.0 (16.3–18.6)	19.7 (17.4–21.5)	*0.0088*	0.79 (0.63–0.93)	*0.025*	0.78 (0.56–1.02)	*0.098*	0.65	65.5

T0, inclusion. T1, T0 + 2 months. T2, end of treatment. OR, odds ratio; aOR, adjusted odds ratio; CI, confidence interval; WBC, white blood cells; C, model C statistic; AIC, Akaike Information Criterion. Only parameters with significant association to the outcome were shown; other tested parameters are available in **Supplementary Table 7**. Slow culture conversion was defined as a persistently positive culture result at T1 followed by a culture conversion at T2. For MIT IFN-γ, associations were calculated for each unit increase. For lymphocyte proportions, associations were calculated for each increase of 5%. ^1^models were adjusted for age, sex, country of origin, drug resistance strain, body mass index at inclusion, and BCG vaccination rate.

In multivariate analyses, significant associations were maintained for MIT IFN-*γ* at T0 (adjusted OR 0.65, p = 0.009), QFT-P IGRA positivity at T0 (aOR 0.045, p = 0.013), and HBHA IGRA positivity at T1 (aOR 0.076, p = 0.045). No significant association was found otherwise (**Supplementary Table 7**). Adjusting the models with neutrophil and monocyte proportions at baseline yielded similar results, but with higher AIC values (**Supplementary Table 8**).

Overall, we observed a slow converter profile including consistent clinical patterns at baseline (low BMI, high neutrophil percentages, low lymphocyte percentages, low TB1 and TB2 IFN-*γ* responses), as well as a downregulated rmsHBHA response at the end of treatment.

## Discussion

In this multicentered prospective study, we assessed the value of QFT-P or rmsHBHA-based IGRAs for pulmonary TB sputum culture conversion monitoring in five cohorts (Bangladesh, Georgia, Lebanon, Madagascar, and Paraguay). We recruited 132 HIV-uninfected culture confirmed pulmonary TB patients, including 28 DR-TB cases. To our knowledge, this is the first time that QFT-P and HBHA IGRAs are prospectively evaluated for treatment monitoring in DS-TB and DR-TB cohorts from high-TB incidence countries.

Consistently with previous works (20, 21), we found that individual monitoring of TB1 and TB2 IFN-*γ* levels during treatment showed little relevancy; we observed important inter-patient heterogeneity, and no significant changes in median values over time. On the contrary, median rmsHBHA IFN-*γ* levels increased significantly throughout treatment, and an increase was observed in most patients. This is consistent with studies associating high rmsHBHA IFN-*γ* levels to latency and controlled infection (23, 25–27), as well as in children (28) and in adults (32) receiving anti-TB treatment. The differences observed between the QFT-P and rmsHBHA IFN-*γ* responses during treatment can be explained by distinct antigen compositions. TB1 and TB2 are peptide pools obtained from secreted antigens, whereas rmsHBHA is a native protein found in mycobacterial cell walls *in vivo*; hence, antigen processing and presentation may differ. Bacterial pathogenesis mechanisms (40) as well as the bactericidal effect of anti-TB treatment could also affect the release of QFT-P and HBHA antigens. In addition, mycobacterial immune escape mechanisms involving HBHA (41, 42) could explain the downregulated *in vitro* IFN-*γ* responses to rmsHBHA during active disease.

Characterization of the association between QFT-P, rmsHBHA IFN-*γ*, and mycobacterial clearance has led us to identify two subsets of conversion rates. In particular, slower culture conversion was associated with QFT-P negativity at T0, consistently with a prior study linking negative or indeterminate QFT-P results with poor treatment outcomes (43), and with HBHA IGRA negativity at T1. More generally, both a general immunosuppression with low non-specific IFN-*γ* (44), and low *M. tuberculosis*-specific IFN-*γ* (45) have been demonstrated during active TB. Thus, an anergic early T-cell-driven response might be involved in slower mycobacterial clearance (43). At the other end of the spectrum, lower levels of IFN-*γ* in slow converters at T2 suggest a link between magnitude of the rmsHBHA-mediated response and mycobacterial clearance.

Our data indicate that rmsHBHA and/or QFT-P IFN-*γ* had low specificity and accuracy compared to the gold standard culture conversion. Because of the small cohort size, this result must be interpreted with caution; but if confirmed, it could suggest that the increase in rmsHBHA IFN-*γ* might be representative of general immune recovery during treatment rather than a specific response to *M. tuberculosis*. Here, this hypothesis is supported by the fact that a low IFN-*γ* response to non-TB specific stimulation (Mitogen tubes) at T0 was also significantly associated with slow culture conversion in multivariate analysis. In addition, immune cross-reactions with HBHA homologs present in environmental mycobacteria have been previously reported (23).

Finally, our study had several limitations. The sample size was relatively small, and patients were included in diverse geographical areas and had different antibiotic regimens. As a consequence, malnutrition levels, untested co-infections (besides HIV and virus B and C hepatitis), different genetic and epigenetic backgrounds, or potential differences in bacterial loads during sputum collection could not be controlled. We were intrigued by differences in IFN-*γ* response to HBHA in the different study sites, which could be linked to ethnic-specific influences over *M. tuberculosis* responses (46). Although adjustment with sociodemographic factors and optimism corrections with a method adapted to small sample sizes (47) were performed, our results need to be confirmed in larger cohorts.

In conclusion, this study described the associations between mycobacterial clearance and immune responses to QFT-P and rmsHBHA IGRAs throughout anti-TB treatment. Lower QFT-P and rmsHBHA IFN-*γ* levels were associated with slower mycobacterial clearance. Our results support a growing body of evidence suggesting that rmsHBHA IFN-*γ* discriminates between the different stages of TB. However, the specificity of both IGRAs was insufficient for treatment monitoring. Further research is needed to clarify how the rmsHBHA response is regulated at the cellular level during treatment, and whether there is any specific interaction with mycobacterial clearance. In particular, evaluating how long rmsHBHA IFN-*γ* values remain stable after treatment would help assess whether it could be a relevant biomarker for relapse prediction.

## Data Availability Statement

The raw data supporting the conclusions of this article will be made available by the authors, without undue reservation.

## Ethics Statement

The studies involving human participants were reviewed and approved by: in Bangladesh, the Research Review Committee and the Ethical Review Committee of icddr, b; in Georgia, the Institutional Review Board of the NTCLD (IORG0009467); in Lebanon, the Institutional Review Board of NINI hospital (IRB-F-01); In Madagascar, the Ministry of Public Health and the Ethical Committee for Biomedical Research (reference number: n°099–MSANP/CERBM); in Paraguay, the Research Ethics Committee and the Scientific Committee of the IICS-UNA (IRB number: IRB00011984; Federalwide Assurance number: FWA00029097). The patients/participants provided their written informed consent to participate in this study.

## Author Contributions

JH is the principal investigator and initiated the project together with DG, NT, SBa, GR, NR, and MH. Data were collected by EK, MU, SBi, CA, PR, CR, PH, JR, AR, and RB. CC performed all the analyses and wrote the manuscript. All authors contributed to the article and approved the submitted version.

## Funding

This work was supported by Fondation Mérieux, Fondation Christophe et Rodolphe Mérieux, and Fondation AnBer, and the grant ANR-18-CE17-0020. A minor part of the study was supported by the Italian Ministry of Health “Ricerca Corrente, Linea 4.”

## Conflict of Interest

MH, MI, and RB had logistic support from the National TB program in Lebanon. DG reports personal fees from Quidel, Qiagen, Janssen, BioMérieux, and Celgene, outside the submitted work. All authors have submitted the ICMJE Form for Disclosure of Potential.
