# Supplementary material for: Relevance of QuantiFERON-TB Gold Plus and Heparin-Binding Hemagglutinin Interferon-γ Release Assays for Monitoring of Pulmonary Tuberculosis Clearance: A Multicentered Study
Source: Front Immunol. 2021 Feb 2;11:616450. doi: 10.3389/fimmu.2020.616450 (PMC7885528; doi:10.3389/fimmu.2020.616450)
Supplement: Supplementary file 2 [file Table_1.docx]

**Supplementary Tables.**

**Supplementary Table 1. Sociodemographic characteristics of the cohort, stratified by country.**

|  | **Bangladesh** | **Georgia** | **Lebanon** | **Madagascar** | **Paraguay** | ***p*** |
| --- | --- | --- | --- | --- | --- | --- |
| N | 38 | 31 | 7 | 36 | 20 |  |
| ***Patient demographics*** |  |  |  |  |  |  |
| Age (years), median (IQR) | 22 (18.25-28)^G,P^ | 34 (28-43) ^B,M^ | 23 (20.5-28.5) | 26 (19.75-36.75) | 28.5 (22.5-38.25)^B^ | ***>0.001*** |
| Sex (male), % (N) | 65.8% (25/38) | 77.4% (24/31) | 42.9% (3/7) | 55.6% (20/36) | 55% (11/20) | *0.23* |
| Drug resistance, % (N) | 44.7% (17/38) ^M,P^ | 32.3% (10/31) ^M^ | 0 | 0^B^ | 5% (1/20)^B^ | ***>0.001*** |
| Treatment failure, % (N) | 2.6% (1/38) | 6.5% (2/31) | 0 | 2.8% (1/36) | 0 | *0.72* |
| BMI at inclusion, median (IQR) | 17.6 (16.3-20.7)^G,L,P^ | 20.3 (18.8-23.8)^B,M^ | 20.9 (20.2-21.2)^B,M^ | 17.1 (16.3-18.2)^G,L,P^ | 20.8 (18.5-22.7)^B,M^ | ***>0.001*** |
| White blood cell absolute count at inclusion (/cumm) | 9500 (8050-10975) | 9800 (7000-12050) | 8610 (6780-12450) | 9370 (5802.5-12002.5) | 11180 (8690-13567.5) | *0.52* |
| Lymphocyte proportion at inclusion (% of WBC) | 20 (18-26.75)^L^ | 18 (15-21.5) | 13.4 (12.55-15.6)^B^ | 17.6 (12.6-24.28) | 18.5 (15.5-23) | ***0.041*** |
| Number of household contacts, median (IQR) | 4.5 (3-7) | 4 (3-4.75) | 5 (4-5.5) | 5 (4-6) | 4 (4-6) | *0.18* |
| BCG vaccination, % (N) | 84.2% (32/38) | 38.7% (12/31) | 14.3% (1/7)* | 91.7% (33/36) | 94.1% (16/17) | ***>0.001*** |
| ***Risk factors and comorbidities*** |  |  |  |  |  |  |
| Smoking, % (N) | 84.2% (32/38) | 38.7% (12/31) | 14.3% (1/7) | 91.7% (33/36) | 50% (10/20) | *0.49* |
| Alcohol abuse, % (N) | 44.7% (17/38)^M^ | 58.1% (18/31)^M,P^ | 28.6% (2/7) | 38.9% (14/36)^B,G^ | 35% (7/20)^G^ | ***0.0010*** |
| Injectable drug use, % (N) | 13.2% (5/38) | 6.5% (2/31) | 0 | 41.7% (15/36) | 5% (1/20) | *0.10* |
| Jail detention history, % (N) | 10.5% (4/38) | 0 | 0 | 0 | 25% (5/20) | ***0****.064* |
| Chronic HCV infection, % (N) | 5.3% (2/38) | 6.5% (2/31) | 14.3% (1/7) | 2.9% (1/34) | 0 | *0.69* |
| Other disease^1^, % (N) | 0 | 3.2% (1/31) | 0 | 2.8% (1/36) | 16.7% (3/18) | *0.060* |
| ***History of TB*** |  |  |  |  |  |  |
| Previous TB, % (N) | 29.7% (11/37) | 10.3% (3/29) | 14.3% (1/7) | 13.9% (5/36) | 20% (4/20) | *0.10* |
| Prior exposure to active TB patients, % (N) | 28.9% (11/38) | 6.7% (2/30) | 42.9% (3/7) | 36.1% (13/36) | 45% (9/20) | *0.36* |
| ***Previous TB outcome*** |  |  |  |  |  |  |
| Cured and completed, % (N) | 42.9% (3/7) | 0 | 0 | 0 | 75% (3/4) | *1* |
| Treatment completed, % (N) | 28.6% (2/7) | 0 | 0 | 0 | 0 | *1* |
| Outcome not evaluated or unknown, % (N) | 0 | 66.7% (2/3) | 0 | 0 | 25% (1/4) | *1* |
| Treatment failure, % (N) | 0 | 33.3% (1/3) | 0 | 33.3% (1/3) | 0 | *1* |

**Footnotes:** BMI: body mass index. IQR: interquartile range. TB: tuberculosis. WBC: white blood cells.

1: asthma, hypertension, inflammation.

Data were compared with Kruskal-Wallis’ test with Dunn’s post-hoc, or Fisher’s test with Bonferroni’s post-hoc when significant.

^B, G ,L, M, P^: initial of study sites that are different from each other (p<0.05). *: different from all other sites.

**Supplementary Table 2. QFT-P or HBHA IFN-y levels in the different study sites.**

| **Parameter** | **Timepoint** | **All** | **Bangladesh** | **Georgia** | **Lebanon** | **Madagascar** | **Paraguay** | ***p (countries)*** |
| --- | --- | --- | --- | --- | --- | --- | --- | --- |
|  |  | N = 132 | N = 38 | N = 31 | N = 7 | N = 36 | N = 20 |  |
| **TB1 [IFN-y] (IU/mL)** | **T0** | 0.52 (0.11-1.78) | 0.49 (0.08-1.9) | 1 (0.24-2.42) | 0.74 (0.33-1.8) | 0.25 (0.02-1.04) | 0.36 (0.24-0.74) | *0.12* |
|  | **T1** | 0.53 (0.1-1.73) | 0.55 (0.18-1.4) | 0.94 (0.17-4.49) | 1.53 (0.53-1.78) | 0.26 (0.05-1.54) | 0.35 (0.11-1.47) | *0.23* |
|  | **T2** | 0.62 (0.12-2.07) | 0.85 (0.17-2) | 0.46 (0.16-1.68) | 0.72 (0.47-2.15) | 0.6 (0.12-2.21) | 0.53 (0.08-1.99) | *0.96* |
| **TB2 [IFN-y] (IU/mL)** | **T0** | 0.64 (0.27-2.06) | 0.55 (0.18-1.98) | 1.51 (0.33-3.88) | 0.62 (0.5-1.76) | 0.56 (0.1-1.29) | 0.53 (0.3-0.91) | *0.24* |
|  | **T1** | 0.66 (0.12-2.91) | 0.84 (0.2-3.6) | 0.77 (0.12-3.83) | 0.92 (0.68-1.62) | 0.48 (0.04-2.08) | 0.37 (0.12-1.7) | *0.47* |
|  | **T2** | 0.82 (0.11-3.43) | 0.9 (0.12-3.29) | 0.61 (0.11-4.17) | 0.87 (0.5-2.55) | 0.8 (0.1-2.33) | 0.59 (0.08-3.75) | *0.92* |
| **MIT [IFN-y] (IU/mL)** | **T0** | 10 (6.47-10) | 10 (7.43-10) | 10 (10-10)^M^ | 7.91 (5.12-10) | 9.44 (1.98-10)^G^ | 10 (5.41-10) | *0.0025* |
|  | **T1** | 10 (8.07-10) | 10 (6.53-10) | 10 (10-10)^M^ | 10 (10-10) | 9.54 (5.35-10)^G^ | 10 (7.72-10) | *0.0075* |
|  | **T2** | 10 (8.59-10) | 10 (7.72-10)^G^ | 10 (10-10)^B,M^ | 10 (9.75-10) | 9.87 (7.42-10)^G^ | 10 (7.09-10) | *0.0095* |
| **HBHA [IFN-y] (IU/mL)** | **T0** | 0.08 (0.01-0.48) | 0.16 (0.03-0.6)^P^ | 0.54 (0.08-4.14)^M,P^ | 0.04 (0.01-0.07) | 0.05 (0.01-0.2)^G^ | 0.05 (0.01-0.07)^B^ | *>0.001* |
|  | **T1** | 0.37 (0.09-1.78) | 0.6 (0.19-1.72)^M,P^ | 2.5 (0.48-8.71)^L,M,P^ | 0.14 (0.07-1.28)^G^ | 0.14 (0.03-0.31)^B,G^ | 0.09 (0.02-0.45)^B^ | *>0.001* |
|  | **T2** | 1 (0.12-5.24) | 5.05 (1.25-10) * | 4.65 (1.46-10) * | 0.36 (0.08-0.92) | 0.08 (0.01-0.31) | 0.5 (0.18-1.09) | *>0.001* |

**Footnotes:** T0: baseline. T1: baseline + 2 months. T2: end of treatment. All values are given after subtraction of NIL [IFN-y].  ^B, G ,L, M, P^: initial of study sites that are different from each other (p<0.05). *: different from all other sites. Data were compared using Kruskal-Wallis’s non-parametric test with Dunn’s post-hoc when significant.

**Supplementary Table 3. Blood count thresholds for stratified IFN-y analysis.**

|  | **T0** | | **T1** | | **T2** | |
| --- | --- | --- | --- | --- | --- | --- |
|  | Q1 | Q3 | Q1 | Q3 | Q1 | Q3 |
| **WBC (cells/mm^3^)** | 7300 | 12000 | 5900 | 9500 | 4900 | 7400 |
| **Neutrophils (% of WBC)** | 70 | 80 | 60 | 70 | 50 | 70 |
| **Lymphocytes (% of WBC)** | 14 | 25 | 20 | 31 | 26 | 37 |

**Footnotes:** T0: baseline. T1: baseline + 2 months. T2: end of treatment. Q1: first quartile. Q3: third quartile. WBC: total white blood cells.

**Supplementary Table 4. Assay performances of the QFT-P and rmsHBHA IGRAs compared to sputum culture.**

| **Test** | **Thresholds** | **Timepoint** | **Sensitivity** | **Specificity** | **Accuracy** |
| --- | --- | --- | --- | --- | --- |
| **Smear microscopy** | *-* | *T1* | 81.1 | 50 | 76.6 |
|  |  | *T2* | 92.9 | 33.3 | 91.5 |
| **QFT-P IGRA** | TB1 ≥ 0.75 IU/mL and TB2 ≥ 0.71 IU/mL | *T1* | 45.3 | 35.3 | 43.8 |
|  |  | *T2* | 45.7 | 66.7 | 46.2 |
| **QFT-P TB2-TB1** | ≥ 0.03 IU/mL | *T1* | 51.6 | 52.9 | 51.8 |
|  |  | *T2* | 54.3 | 66.7 | 54.6 |
| **HBHA IGRA** | ≤ 0.22 IU/mL | *T1* | 64.2 | 64.7 | 64.3 |
|  |  | *T2* | 66.9 | 0 | 65.4 |
| **HBHA and QFT-P IGRA** | *-* | *T1* | 86.3 | 23.5 | 76.8 |
|  |  | *T2* | 82.7 | 0 | 80.8 |
| **HBHA and TB2-TB1** | *-* | *T1* | 80 | 35.3 | 73.2 |
|  |  | *T2* | 85 | 0 | 83.1 |

**Footnotes:** T0: baseline. T1: baseline + 2 months. T2: end of treatment.

For all IGRA variables, cutoffs adapted for this study on active TB patients were calculated using AUC analyses. Respective cutoffs are indicated in the “Threshold” column. The overall QFT-P test was considered positive if either TB1 or TB2 were above the indicated thresholds. The “HBHA and QFT-P IGRA” variable was defined as follows: positive when HBHA-IGRA results are negative and QFT-P results are positive; negative when HBHA-IGRA results are positive and/or QFT-P results are negative or indeterminate. The “HBHA and TB2-TB1” variable was defined as follows: positive when HBHA-IGRA results are negative and TB2-TB1 is strictly greater than the indicated threshold; negative when HBHA-IGRA results are positive and/or TB2-TB1 is equal to or lesser than the indicated threshold.

**Supplementary Table 5. Associations between time to culture conversion and WBC counts.**

|  |  | **Successfully treated patients with available T1 culture results (n = 112)** | | | |
| --- | --- | --- | --- | --- | --- |
|  | **Timepoint** | **Fast converters** | **Slow converters** | **Failure or relapse** | ***p*** |
| **N** |  | 92 | 16 | 4 |  |
| Absolute WBC count (per mm^3^) | *T0* | 9500 (7302.5-11425) | 9385 (7822.5-14325) | 12200 (12082.5-13400) | *0.077* |
|  | *T1* | 7535 (6247.5-9367.5) | 8500 (6220-10900) | 10780 (9470-11300) | *0.14* |
|  | *T2* | 6190 (4742.5-7812.5) | 6750 (4387.5-7687.5) | 6050 (5595-7247.5) | *0.97* |
| Neutrophil % of WBC | *T0* | 75 (68-79) | 75 (71.97-78.25) | 84 (81.5-86.5)* | ***0.022*** |
|  | *T1* | 67.55 (60-72.17) | 67.5 (61.1-75) | 79 (75-81.75)* | ***0.043*** |
|  | *T2* | 60.15 (54.75-68) | 58.5 (51.75-66.97) | 64.5 (59-71.75) | *0.49* |
| Lymphocyte % of WBC | *T0* | 19 (15-26) | 17.5 (12.8-19.5) | 12.5 (9.2-15.2)* | ***0.017*** |
|  | *T1* | 25 (20.7-31) | 23 (16.2-28.0) | 15.5 (11-21.2)* | ***0.027*** |
|  | *T2* | 30 (25.9-36) | 29.5 (23.5-36.2) | 21 (17.7-26.5) | *0.21* |
| High absolute WBC count (>3rd quartile) | *T0* | 21.7% (20/92) | 31.2% (5/16) | 75% (3/4) | *0.050* |
|  | *T1* | 20.7% (19/92) | 43.8% (7/16) | 75% (3/4)* | ***0.014*** |
|  | *T2* | 29.3% (27/92) | 25% (4/16) | 25% (1/4) | *1* |
| High neutrophil % (>3rd quartile) | *T0* | 22.8% (21/92) | 18.8% (3/16) | 100% (4/4)* | ***0.0053*** |
|  | *T1* | 23.9% (22/92) | 31.2% (5/16) | 75% (3/4) | *0.081* |
|  | *T2* | 28.3% (26/92) | 31.2% (5/16) | 50% (2/4) | *0.62* |
| Low lymphocyte % (<1st quartile) | *T0* | 14.1% (13/92) | 37.5% (6/16) | 50% (2/4)* | ***0.020*** |
|  | *T1* | 19.6% (18/92) | 37.5% (6/16) | 50% (2/4) | *0.12* |
|  | *T2* | 22.8% (21/92) | 31.2% (5/16) | 75% (3/4) | *0.06* |

**Footnotes:** Data are given as median (interquartile range) or % (N). WBC: white blood cells. T0: baseline. T1: baseline + 2 months. T2: end of treatment. Fast converters: culture conversion between T0 and T1. Slow converters: culture conversion between T1 and T2. Treatment failure: positive culture at T2 or T3 (end of treatment + 2 months). *: significantly different from both other groups (Kruskal-Wallis test + Dunn’s post-hoc test).

**Supplementary Table 6. Sociodemographic characteristics and culture conversion profile**

|  | **Fast converters** | **Slow converters** | **Failure or relapse** | ***p*** |
| --- | --- | --- | --- | --- |
| **N** | **92** | **16** | **4** |  |
| ***Patient demographics*** |  |  |  |  |
| Age (years), median (IQR) | 26.5 (21-36.25) | 33.5 (25.75-47.75) | 38 (30.5-48.25) | *0.099* |
| Sex (male), % (N) | 63% (58/92) | 62.5% (10/16) | 100% (4/4) | *1* |
| Drug resistance, % (N) | 26.1% (24/92) | 18.8% (3/16) | 0 | *0.76* |
| Country of origin, % (N) |  |  |  |  |
| *Bangladesh* | 38% (35/92) | 18.8% (3/16) | 0 | *0.14* |
| *Georgia* | 26.1% (24/92) | 25% (4/16) | 50% (2/4) | *0.93* |
| *Lebanon* | 5.4% (5/92) | 0 | 0 | *0.34* |
| *Madagascar* | 10.9% (10/92)* | 56.2% (9/16)* | 25% (1/4) | ***>0.001*** |
| *Paraguay* | 19.6% (18/92) | 0 | 25% (1/4) | *0.053* |
| BMI at inclusion, median (IQR) | 19.7 (17.3-21.4)* | 17.0 (16.2-18.6)* | 17.5 (15.9-20.2) | ***0.0088*** |
| White blood cell absolute count at inclusion (/mm^3^) | 9500 (7302.5-11425) | 9385 (7822.5-14325) | 12200 (12082.5-13400) | *0.75* |
| Lymphocyte proportion at inclusion (% of WBC) | 19 (15-26) | 17.5 (12.8-19.55) | 12.5 (9.25-15.25) | *0.099* |
| Number of household contacts, median (IQR) | 4 (3-6) | 3.5 (3-5.25) | 5.5 (4.75-8) | *0.44* |
| BCG vaccination, % (N) | 83.8% (62/74) | 100% (13/13) | 100% (2/2) | *0.19* |
| ***Risk factors and comorbidities*** |  |  |  |  |
| Smoking, % (N) | 42.4% (39/92) | 43.8% (7/16) | 100% (4/4) | *1* |
| Alcohol abuse, % (N) | 17.4% (16/92) | 12.5% (2/16) | 50% (2/4) | *0.32* |
| Injectable drug use, % (N) | 4.4% (4/91) | 0 | 0 | *1* |
| Jail detention history, % (N) | 7.7% (7/91) | 6.2% (1/16) | 50% (2/4) | *1* |
| Chronic HCV infection, % (N) | 1.4% (1/71) | 8.3% (1/12) | 0 | *0.27* |
| Other disease^1^, % (N) | 5% (4/80) | 8.3% (1/12) | 0 | *0.51* |
| ***History of TB*** |  |  |  |  |
| Previous TB, % (N) | 17.3% (16/92) | 12.5% (2/16) | 25% (1/4) | *0.73* |
| Prior exposure to active TB patients, % (N) | 26.4% (24/91) | 12.5% (2/16) | 75% (3/4) | *1* |

**Footnotes:** BMI: body mass index. IQR: interquartile range. TB: tuberculosis. WBC: white blood cells.

1: asthma, hypertension, inflammation. Data were compared with the Kruskal-Wallis test and Dunn’s post hoc, or Fisher’s test. *: groups significantly different from each other.

**Supplementary Table 7. Associations between time to culture conversion and IFN-y response.**

| **Parameter** | **Timepoint** | **Univariate analysis** | | **Multivariate analysis^1^** | | | |
| --- | --- | --- | --- | --- | --- | --- | --- |
|  |  | **OR (95%CI)** | ***p*** | **aOR (95%CI)** | ***p*** | **C** | **AIC** |
| TB1 IFN-y | *T0* | 0.87 (0.611 - 1.09) | *0.32* | 0.914 (0.652 - 1.213) | *0.55* | 0.62 | 67.1 |
|  | *T1* | 0.879 (0.646 - 1.08) | *0.30* | 1.051 (0.74 - 1.392) | *0.74* | 0.61 | 67.4 |
|  | *T2* | 1.01 (0.831 - 1.18) | *0.91* | 1.365 (1.002 - 1.943) | *0.058* | 0.66 | 63.6 |
| TB2 IFN-y | *T0* | 0.81 (0.543 - 1.03) | *0.17* | 0.856 (0.592 - 1.143) | *0.33* | 0.65 | 66.4 |
|  | *T1* | 0.874 (0.659 - 1.07) | *0.25* | 0.992 (0.715 - 1.289) | *0.96* | 0.62 | 67.5 |
|  | *T2* | 0.99 (0.818 - 1.15) | *0.90* | 1.162 (0.911 - 1.493) | *0.22* | 0.62 | 66.0 |
| HBHA IFN-y | *T0* | 0.336 (0.023 - 0.916) | *0.25* | 0.241 (0.004 - 1.068) | *0.36* | 0.68 | 64.8 |
|  | *T1* | 0.989 (0.814 - 1.15) | *0.89* | 1.068 (0.746 - 1.515) | *0.69* | 0.61 | 67.3 |
|  | *T2* | 0.843 (0.674 - 0.993) | *0.072* | 0.983 (0.712 - 1.333) | *0.91* | 0.64 | 67.5 |

**Footnotes:** T0: inclusion. T1: T0 + 2 months. T2: end of treatment. OR: odds ratio. aOR: adjusted odds ratio. CI: confidence interval. WBC: white blood cells. C: model C statistic. AIC: Akaike Information Criterion. Slow culture conversion was defined as a persistently positive culture result at T1 followed by a culture conversion at T2. TB1, TB2 and HBHA IFN-y levels were measured in IU/mL. For continuous independent variables, associations were calculated for each unit increase. 1: models were adjusted for age, sex, country of origin, drug resistance strain, body mass index at inclusion, and BCG vaccination rate.

**Supplementary Table 8. Associations between time to culture conversion and IFN-y response, adjusted for neutrophil and monocyte proportions at baseline.**

| **Parameter** | **Timepoint** | **Multivariate analysis^1^** | | | |
| --- | --- | --- | --- | --- | --- |
|  |  | **aOR (95% CI)** | ***p*** | **C** | **AIC** |
| TB1 IFN-y | *T0* | 0.939 (0.642 - 1.34) | *0.73* | 0.62 | 68.0 |
|  | *T1* | 0.957 (0.636 - 1.32) | *0.80* | 0.61 | 68.0 |
|  | *T2* | 1.42 (1.027 - 2.08) | *0.054* | 0.68 | 63.6 |
| TB2 IFN-y | *T0* | 0.844 (0.563 - 1.206) | *0.36* | 0.64 | 67.2 |
|  | *T1* | 0.928 (0.64 - 1.247) | *0.64* | 0.62 | 67.8 |
|  | *T2* | 1.19 (0.927 - 1.57) | *0.16* | 0.67 | 66.1 |
| HBHA IFN-y | *T0* | 0.341 (0.006 - 1.185) | *0.45* | 0.66 | 66.4 |
|  | *T1* | 1.133 (0.779 - 1.661) | *0.49* | 0.62 | 67.6 |
|  | *T2* | 1.004 (0.723 - 1.39) | *0.98* | 0.62 | 68.1 |
| MIT IFN-y | *T0* | 0.62 (0.391 - 0.857) | *0.013* | 0.74 | 58.7 |
|  | *T1* | 0.711 (0.498 - 0.954) | *0.034* | 0.70 | 62.8 |
|  | *T2* | 0.799 (0.555 - 1.122) | *0.19* | 0.63 | 66.3 |
| Positive QFT-P IGRA | *T0* | 0.045 (0.002 - 0.404) | *0.022* | 0.75 | 59.5 |
|  | *T1* | 0.279 (0.036 - 1.631) | *0.17* | 0.66 | 66.1 |
|  | *T2* | 2.69 (0.462 - 21.2) | *0.29* | 0.66 | 66.9 |
| Positive HBHA IGRA | *T0* | 0.551 (0.057 - 4.366) | *0.57* | 0.61 | 67.8 |
|  | *T1* | 0.075 (0.003 - 0.689) | *0.045* | 0.72 | 62.7 |
|  | *T2* | 0.623 (0.098 - 4.45) | *0.62* | 0.61 | 67.8 |
| Lymphocyte % of WBC | *T0* | 0.89 (0.482 - 1.685) | *0.71* | 0.61 | 67.9 |
|  | *T1* | 0.898 (0.788 - 0.988) | *0.055* | 0.66 | 63.0 |
|  | *T2* | 1.00 (0.915 - 1.09) | *0.98* | 0.61 | 68.1 |
| Body mass index | *T0* | 0.912 (0.578 - 1.35) | *0.66* | 0.64 | 66.1 |

**Footnotes**: T0: inclusion. T1: T0 + 2 months. T2: end of treatment. aOR: adjusted odds ratio. CI: confidence interval. WBC: white blood cells. C: model C statistic. AIC: Akaike Information Criterion. Slow culture conversion was defined as a persistently positive culture result at T1 followed by a culture conversion at T2. TB1, TB2 and HBHA IFN-y levels were measured in IU/mL. 1: models were adjusted for age, sex, country of origin, drug resistance strain, body mass index at inclusion, BCG vaccination rate, and neutrophil and monocyte proportion at baseline.

**Supplementary Figure**

**
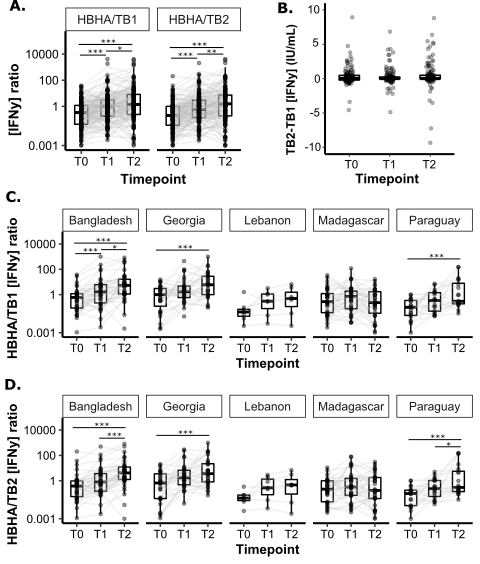
**

**Supplementary Figure 1. Dynamics of plasmatic IFN-y response to QFT-P and HBHA stimulations over the course of TB therapy.** Data are given as median + interquartile range. **A.** Evolution of the HBHA/TB1 and HBHA/TB2 IFN-y ratios throughout treatment. **B.** Evolution of the TB2-TB1 IFN-y response (QFT-P CD8+ T cell response) throughout treatment. Stratification per study site of the HBHA/TB1 (**C.**) and HBHA/TB2 (**D.**) ratios. Bangladesh (n = 38), Georgia (n = 31), Lebanon (n = 7), Madagascar (n = 36), Paraguay (n = 20). Each black dot represents one patient at one timepoint. Grey lines connect data points from a same patient. T0: baseline. T1: baseline + 2 months. T2: end of treatment. Data were compared using Friedman’s Exact Test with the Wilcoxon-Nemenyi-McDonald-Thompson post-hoc, or the Mann-Whitney U test (panel B). *: p<0.05; **: p<0.01; ***: p<0.001.
